# Bayesian Gaussian regression analysis of malnutrition for children under five years of age in Ethiopia, EMDHS 2014

**DOI:** 10.1186/s13690-018-0264-6

**Published:** 2018-03-26

**Authors:** Seid Mohammed, Zeytu G. Asfaw

**Affiliations:** 1 Department of Statistics, Aksum University, Aksum, Ethiopia; 2 School of Mathematical and Statistical Sciences, College of Natural and Computational Sciences, Hawassa University, Hawassa, Ethiopia

**Keywords:** Children, Malnutrition, Gaussian linear model, Bayesian approach, BayesX

## Abstract

**Background:**

The term malnutrition generally refers to both under-nutrition and over-nutrition, but this study uses the term to refer solely to a deficiency of nutrition. In Ethiopia, child malnutrition is one of the most serious public health problem and the highest in the world. The purpose of the present study was to identify the high risk factors of malnutrition and test different statistical models for childhood malnutrition and, thereafter weighing the preferable model through model comparison criteria.

**Methods:**

Bayesian Gaussian regression model was used to analyze the effect of selected socioeconomic, demographic, health and environmental covariates on malnutrition under five years old child’s. Inference was made using Bayesian approach based on Markov Chain Monte Carlo (MCMC) simulation techniques in BayesX.

**Results:**

The study found that the variables such as sex of a child, preceding birth interval, age of the child, father’s education level, source of water, mother’s body mass index, head of household sex, mother’s age at birth, wealth index, birth order, diarrhea, child’s size at birth and duration of breast feeding showed significant effects on children’s malnutrition in Ethiopia. The age of child, mother’s age at birth and mother’s body mass index could also be important factors with a non linear effect for the child’s malnutrition in Ethiopia.

**Conclusions:**

Thus, the present study emphasizes a special care on variables such as sex of child, preceding birth interval, father’s education level, source of water, sex of head of household, wealth index, birth order, diarrhea, child’s size at birth, duration of breast feeding, age of child, mother’s age at birth and mother’s body mass index to combat childhood malnutrition in developing countries.

**Electronic supplementary material:**

The online version of this article (10.1186/s13690-018-0264-6) contains supplementary material, which is available to authorized users.

## Background

Malnutrition remains one of the most common causes of morbidity and mortality among under five years old children throughout the World [1]. Worldwide, over 10 million children under the age of 5 years die every year from preventable and treatable illnesses despite effective health interventions. At least half of these deaths are caused by malnutrition. The 2011 Ethiopian DHS report shows that 29% of children under age five are underweight (have low weight-for-age), and 9% are severely underweight.

**Fig. 1:**
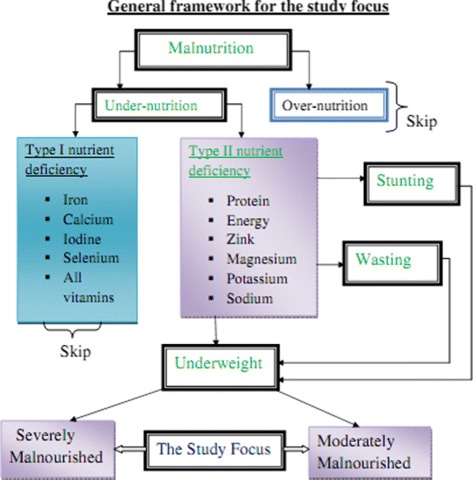
General framework for the study on under five years old children malnutrition, EMDHS 2014

The term “malnutrition” is sometimes also used synonymously for undernutrition. However, strictly speaking, malnutrition includes both undernutrition as well as over nutrition, Fig. 1. Undernutrition may be defined as insufficient intake of energy and nutrients to meet an individual’s needs to maintain good health [2, 3]. Undernutrition is classified into type I and type II nutrient deficiencies [4]. In this paper, we have concerned on the type II nutrient deficiencies. Type II nutrients include protein, energy, zinc, magnesium, potassium and sodium. When there is a deficiency in one of the type II or growth nutrients, the person stops growing [5].

There are three kinds of type II undernutrition in children: stunting, underweight and wasting [6]. In nutrition, anthropometric data collected in the Ethiopian mini demographic and health survey (EMDHS) are used to calculate three indices of nutritional status such as height-for-age, weight-for-age and weight-for-height. These three indices are measured through Z-scores. Z-scores represents the number of standard deviations by which an individual child’s anthropometric index differs from the median of the World Health Organization international growth reference population [7].

Weight-for-age (Underweight) is a composite index of height-for-age (Stunted) and weight-for-height (Wasted). A child can be underweight for his/her age because he or she is stunted, wasted, or both. Weight-for-age is an overall indicator of a population’s nutritional health. Children with weight-for-age Z-scores below minus two standard deviations from the median of the reference population are considered as underweight. Furthermore, children with Z-scores below minus three standard deviations from the median of the reference population are considered to be severely underweight, while children with Z-scores between minus three and minus two standard deviations are known to be moderately underweight [8].

Weight-for-age value for a child *i* is determined using a Z-score (*Z*_*i*_) which is defined as:

$$\begin{array}{*{20}l} Z_{i}= \frac{{AI}_{i} - MAI}{\sigma} \end{array} $$

where *AI*_*i*_ represents child’s anthropometric indicator (weight at a certain age) for the *i*^*th*^ child, *i*=1,2,....*n*, *MAI* is median of the reference population and *σ* is standard deviation (SD) of the reference population.

Authors are interested in modeling the various possible factors and their contribution for the high prevalence of malnutrition problems. To expand authors understanding of the most common and consistent factors on the risk of childhood malnutrition, it is necessary to consider expected determinants for malnutrition using Bayesian approach. Thus, the present study focuses on the identification of the high risk factors of malnutrition and test different statistical models for childhood malnutrition and, thereafter weighing the preferable model through model comparison criteria.

## Methods

### Study sample and setting

The data sets used in the present study were obtained from the Ethiopian Mini Demography Health Survey, EMDHS (2014). The survey drew a representative sample of women of reproductive age (15-49), by administering a questionnaire and making an anthropometric assessment of women and their children that were born within the previous five years [9].

For the 2014 EMDHS, a representative sample is approximately 4893 children aged less than 59 months with complete anthropometric measurements of underweight [8]. In the present study, data are presented for 3115 of these children considering that values had missed for malnutrition (underweight) as well as it’s determinants.

### Study variables

The causes of children malnutrition are multiple. Our analysis started with a large number of covariates including a set of socio-economic, demographic, health and environmental characteristics that are considered as the most important determinants of children’s malnutrition as suggested by previous studies ([10–12]).

#### Response variable

In our application, malnutrition (underweight) was considered as the response variable. Z-score (in a standardized form) was used as a continuous variable to maximize the amount of information available in the data set.

#### Explanatory variables

We have considered both continuous and categorical variables as expected determinants of children malnutrition.

**Continuous covariates**

Child’s age in months (Chag)

Mother’s age at birth (MAB)

Mother’s body mass index (BMI)

**Categorical Covariates (as factor coding)**

Sex of child (Chsex: female or male)

Mother’s current work status (MWsts: no or yes)

Mother’s education level (MED: no formal education, primary or secondary and above)

Father’s education level (FED: no formal education, primary or secondary and above)

Locality where child lives (Residence: rural or urban)

Wealth index (Welnx: poor, medium or rich)

Duration of breast feeding (Brstfdg: never breast fed, fed but no currently breast feeding or still breast feeding)

Sex of household head (HHsex: female or male)

Age of household head in years (HHage: 15-38, 39-63 or above 63)

Birth order (Border: 1-4, 5-9 or 10 and above)

Preceding birth interval in months (PresBint: less than 24, 24-47 or 48 and above)

Child’s size at birth (Chsize: small, average or large)

Sources of drinking water (Water: not improved or improved)

Toilet facility (Toilet: no facility or have facility)

Had diarrhea recently (Diarhea: no or yes)

Ever had vaccination (Vacination: no or yes)

Whether mother take drug for intestinal parasites during pregnancy (Drug: no or yes)

## Statistical models

The statistical analysis employed in the present study is based on Bayesian approaches which allow a flexible framework for realistically complex models. These approaches allow us to analyze usual linear effects of categorical covariates and non linear effects of continuous covariates within a unified semi-parametric Bayesian framework for modeling and inference. Basically, we are interested in model fitting of Gaussian linear regression model to identify those variables which have linear effects on the children’s malnutrition. Extending to additive Gaussian regression model to find out those variables which have non linear effect on children malnutrition. Moreover, we have considered the semi-parametric regression model to look at both effects. Finally, we employed the model comparison Criterion to choose the preferable model for the data analysis.

### Gaussian linear regression model

Consider the normal linear regression model in which a response variable *y* is related to one or more explanatory variables. For a random sample of *n* individuals, the model becomes:

1 $$ \eta_{i} =W_{i}^{'}\nu + V_{i}^{'}\gamma  $$

Here, *W*_*i*_=(*w*_*i*1_,....,*w*_*ip*_) is a vector of continuous covariates. *ν*=*ν*_1_,.....,*ν*_*p*_ is a vector of regression coefficients for the continuous covariates. *V*_*i*_=(*v*_*i*1_,....,*v*_*ik*_) is a vector of categorical covariates. *γ*=*γ*_0_,*γ*_1_,.....,*γ*_*k*_ is a vector of regression coefficients for the categorical covariates. *p*=1,2,3;*k*=1,2,....,17 and *i*=1,2,...,3115.

And also, this model can be written as:

$$\eta_{i}=X_{i}\beta $$

where: *X*_*i*_=(*W*_*i*_,*V*_*i*_) and *β*=(*ν*,*γ*).

### Gaussian semi-parametric regression model

The assumption of a parametric linear predictor for assessing the influence of covariate effects on responses seems to be rigid and restrictive in practical application situation and also in many real statistically complex situation since their forms can not be predetermined a priori. Besides, practical experience has shown that continuous covariates often have nonlinear effects. In our study, for the continuous covariates in the data set, the assumption of a strictly linear effect on the predictor may not be appropriate, i.e. some effects may be of unknown nonlinear form (such as, mother’s age and mother’s BMI) as suggested by Khaled [12] and Mohammed [13].

Hence, it is necessary to seek for a more flexible approach for estimating the continuous covariates by relaxing the parametric linear assumptions. This in turn allows continuous covariates to follow their true functional form. This can be done using an approach referred to as nonparametric regression model. To specify a non parametric regression model, an appropriate smooth function that contains the unknown regression function needs to be chosen.

The semi-parametric regression model is obtained by extending model (1) as follows:

2 $$ \eta_{i} = f_{1}(w_{i1})+....+f_{p}(w_{ip}) + V_{i}^{'}\gamma  $$

Here, *i*=1,2,...,*n* and *p*=3*f*_*i*_(*w*_*i*_) are smooth functions of the continuous covariates and $V_{i}^{'}\gamma $ represents the strictly linear part of the predictor.

### Bayesian inference

It is based on the posterior distribution. Basic statistics like mean, mode, median, variance and quartiles are used to characterize the posterior distribution. The joint conjugate prior for (*β*,*σ*^2^) has the structure [14]:

$$p\left(\beta,\sigma^{2}\right)= p\left(\beta|\sigma^{2}\right) p\left(\sigma^{2}\right) $$

Then, the posterior distribution is given by:

3 $$ p\left(\beta,\sigma^{2}|y\right) \propto p\left(y|\beta,\sigma^{2}\right) p\left(\beta|\sigma^{2}\right)p\left(\sigma^{2}\right)  $$

where the conditional prior for the parameter vector *β* is the multivariate Gaussian distribution with mean $\hat {\beta }$ and covariance matrix *σ*^2^*V*_*β*_ [14]:

$$\beta|\sigma^{2} \sim N_{p}\left(\hat{\beta}, \sigma^{2} V_{\beta}\right) $$

and to obtain the prior for *σ*^2^, now we integrate *β* out of the joint posterior to get the marginal posterior for *σ*^2^ [14]:

$$\pi\left(\sigma^{2}\right)= \int \pi\left(\beta,\sigma^{2}\right) d\beta $$

Then, the marginal posterior distribution of *σ*^2^ becomes inverted gamma, which is clearly

$$IG(a, b) $$

In Bayesian approach, the vector of unknown parameters to be estimated is *θ*=(*β*,*σ*^2^). Therefore, we need to choose prior distributions for these parameters. If prior information is scarce, a large value for the variance parameter should be chosen, so that the prior distribution is flat. This type of prior is called non informative prior. On the other hand, if the analyst has considerable information about the coefficient *β*, he/she should choose a small value for the variance parameter.

For our specific application in model (1), due to the absence of any prior knowledge we use a noncommittal or vague priors *π*(*ν*)∝*constant* and *π*(*γ*)∝*constant* for the parameters of fixed (linear) effects. For each regression coefficient, the prior distribution is a very broad normal distribution, with a mean of zero and a standard deviation that is extremely large relative to the scale of the data. The same assumption is made for the prior on the intercept. Finally, the prior on the standard deviation of the predicted value is merely a uniform distribution extending from zero to an extremely large value far beyond any realistic value for the scale of the data. In the specific analysis demonstrated in this section of our article, the data were standardized so that the prior would be broad regardless of the original scale of the data. The results were then simply algebraically transformed back to the original scale. For the standardized data, the prior on the intercept and regression coefficients was a normal distribution with mean at zero and large standard deviation (example; 1000). This normal distribution is virtually flat over the range of possible intercepts and regression coefficients for standardized data.

To begin, we will choose a non-informative (vague) prior [14]. But in model (2), the parameters of interest *f*_*j*_ is considered as random variables and have to be supplemented with appropriate prior assumptions. Several alternatives are available as smoothness priors for the unknown functions *f*_*j*_(*w*_*j*_). Among the others, random walk priors [14], Bayesian Penalized-Splines [15], Bayesian smoothing splines [16] are the most commonly used. In the present study, the Bayesian smoothing spline was used by taking cubic P-spline with second order random walk priors [17, 18].

By defining an additional hyperprior for the variance parameters the amount of smoothness can be estimated simultaneously with the regression coefficients. We assign the conjugate prior for $\tau ^{2}_{j}$ which is an inverse gamma prior with hyper parameters *a*_*j*_ and *b*_*j*_, i.e $\tau ^{2}_{j} \sim IG(a_{j}, b_{j})$. Common choices for *a*_*j*_ and *b*_*j*_ are *a*_*j*_ = 1 and *b*_*j*_ small, e.g. *b*=0.005*orb*_*j*_=0.0005. Alternatively we may set *a*_*j*_=*b*_*j*_, e.g. *a*_*j*_=*b*_*j*_=0.001. Based on experience from extensive simulation studies the researcher use *a*_*j*_=*b*_*j*_=0.001 as the standard choice. Since the results may considerably depend on the choice of *a*_*j*_ and *b*_*j*_ some sort of sensitivity analysis is strongly recommended. For instance, the models under consideration could be re-estimated with (a small) number of different choices for *a*_*j*_ and *b*_*j*_.

### Model comparison and selection

Model selection is the task of selecting the best model from a set of candidate models based on the performance of each model.

The next question is why should we consider model selection? There are several reasons. First, people tend to believe or can understand simpler models with fewer predictors and less complicated structure. Second, one can certainly add more and more features into the model without screening and get better and better fit, till perfect fit, but the problem is over fitting. Note that the authors want to find the best-predicting model not the best fitting model.

Model comparison is required for a diversity of activities, including variable selection in regression, determination of the number of components or the choice of parametric family. In frequentest approach, we can also perform the familiar statistical test via the *anova* function. As with frequentest analogues, Bayesian model comparison will not inform about which model is true, but rather about the preference for the model given the data and other information [14].

The models proposed in the present study are quite general and the model building process can be quite challenging. Currently, an automated procedure for Bayesian model selection is not available. However, a few recommendations are possible:

- Users should try to incorporate everything that is theoretically possible.
- Different Bayesian models could be compared via the Deviance Information Criterion (DIC) [19].

In the present study, AIC (Akaike Information Criterion) is used to compare the linear frequent and the linear Bayesian approach. Then, we compared the additive frequent and the Bayesian approach by using the GCV (Generalized Cross-Validation) score.

The classical approach to model comparison involves a trade-off between how well the model fits the data and the level of complexity. Spiegelhalter et al. [19] devised a selection criterion which was based on Bayesian measures of model complexity and how good a fit the model is for the data. The measure of complexity which we adopted in this work is suggested by [19].

**Fig. 2:**
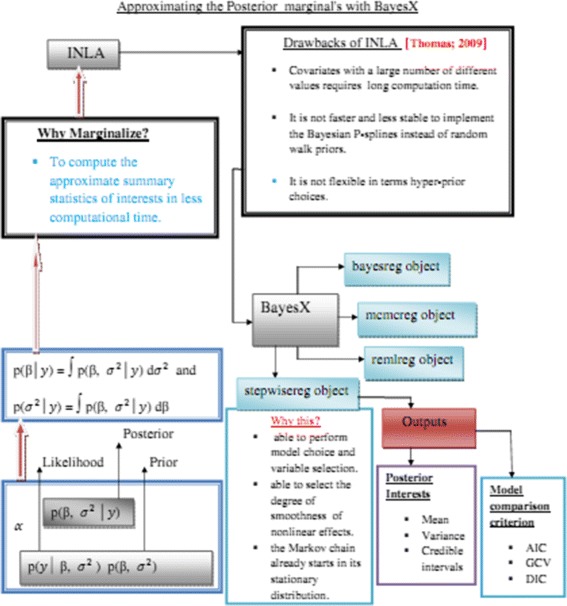
Chart that approximating the Posterior marginal distribution through BayesX

A widely used statistic for comparing models in a Bayesian framework is the DIC. DIC is a hierarchical modeling generalization of the AIC (Akaike information criterion) and BIC (Bayesian information criterion). It is particularly useful in Bayesian model selection problems where the posterior distributions of the models have been obtained by Markov chain Monte Carlo (MCMC) simulation. The idea is that models with smaller DIC should be preferred to models with large DIC, Fig. 2.

## Results

### Descriptive analysis

**Fig. 3:**
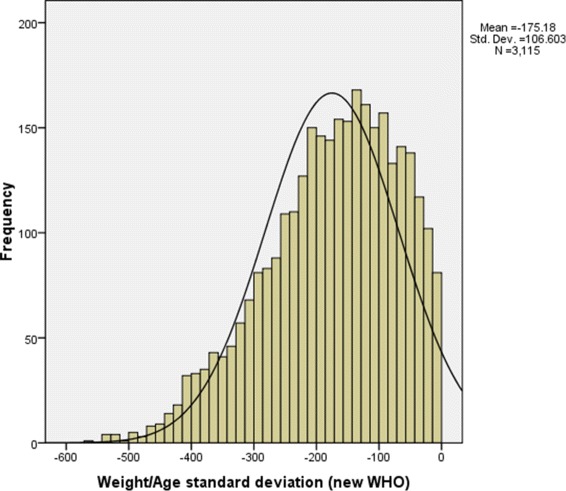
Histogram for underweight showing a normal distribution in under five years old children malnutrition, EMDHS 2014

**Fig. 4:**
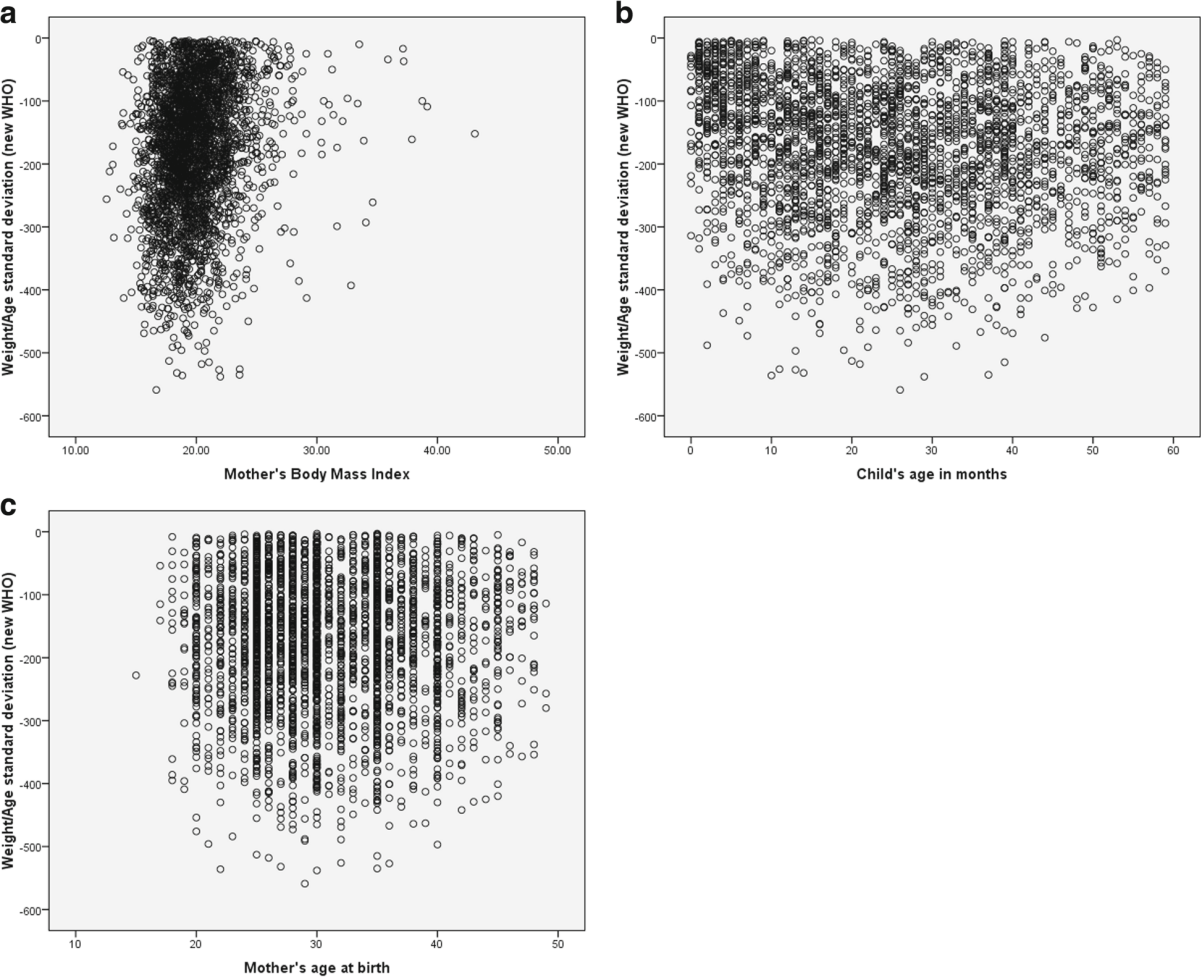
Scatter plots that represent the relationship between each continuous covariates with under five years old children malnutrition, EMDHS 2014

**Fig. 5:**
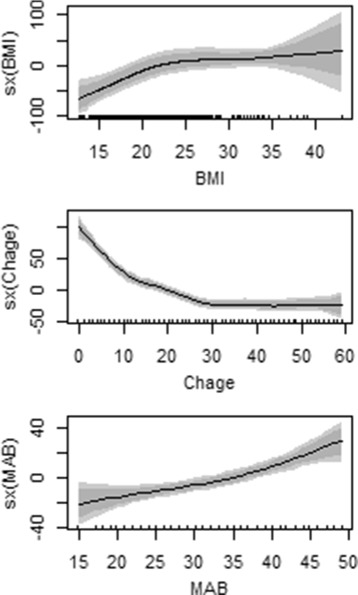
The Non Linear Effects of Continuous Variables on under five years old children malnutrition, EMDHS 2014

In the present study, the response variable malnutrition seems reasonable to assume at least approximately Gaussian (normal) distributed since it has a continuous Z-score value. Then it can be reasonably approximated by a Gaussian distribution that can be observed from the histogram plot in Fig. 3 and Additional file 1. In Fig. 4, the scatter plot of malnutrition vs each continuous covariates such as child age in months, mother’s age at birth and mother’s body mass index showed that there is no definite pattern of relationships respectively. To overcome this problem, we deployed a non parametric method to explore relationships among covariates (see Fig. 5).

The main purpose of the present descriptive analysis was to describe the variation among the categorical explanatory variables with regard to children malnutrition in Ethiopia through percentage value.

Table 1 showed that the proportion of children’s malnutrition decreases as the age of head of household, child’s birth order and father’s as well as mother’s education level increases. The proportion of underweight children is approximately nine times higher for those born to uneducated father’s than for those whose father’s have more than secondary education (59.3% versus 6.7%). Children born from mothers in the poorest wealth quintile are more than twice as likely to be malnourished as children born from mothers in the richest wealth quintile (57.3% compared with 26.1%). The proportion of children malnutrition, as can be seen in Table 1, differs by type of place of residence: urban and rural. From Table 1 we observed that children reside in rural areas were more likely to be malnourished. On the other hand, children ever had vaccination were apparently more often affected by malnutrition than those never got vaccination but there was no consistent trend in the pattern of malnutrition with respect to children got vaccination. With regards to underweight children, female children are slightly more likely to be malnourished than male children (52% versus 48%).

**Table 1:** Distribution of categorical variables vs under five years old children malnutrition, EMDHS 2014

Factors	Category	Percentage value (%)
Vaccination	No	26.9
	Yes	73.1
Fathers education level	No formal education	59.3
	Primary	34
	Secondary and above	6.7
Birth order	1-4	51.2
	5-9	43.2
	10+	5.6
Place of residence	Rural	90.1
	Urban	9.9
Wealth index	Poor	57.3
	Medium	16.6
	Rich	26.1
Age of household head	15-38	57.1
	39-63	38.1
	64+	4.8
Preceding birth interval	< 24	21
	24-47	54.9
	48+	24.1
Mothers education level	No formal education	77.7
	Primary	20.6
	Secondary and above	1.7
Child size at birth	Small	34.8
	Average	38.5
	Large	26.7
Child sex	Female	48
	Male	52

Regarding Child’s birth interval in month, the lowest prevalence of all child’s underweight status was observed among children whose birth interval is less than 24 months (21%), Table 1. As opposed to the highest prevalence of all child’s underweight status was recorded from children whose birth interval is between 24 and 47 (54.9%). Also, children reported as small or average at birth are much more likely to be malnourished (34.8% and 38.5%, respectively) than those reported as large at birth (26.7%).

### Inferential analysis

In this section, the statistical procedure was used in combination with the BayesX stepwise selection method. This enabled us to select different covariates which contribute to malnutrition. Table 2 gives results for the fixed effects on the malnutrition of children under five years old in Ethiopia. The output gives posterior means, posterior median along with their standard deviations and 95% credible intervals.

**Table 2:** Results of fixed effects estimation results of parametric coefficients

	Mean	Sd	2.5%	50%	97.5%
(Intercept)	-201.1820	13.6497	-228.1570	-201.3500	-174.6240
Father education_1_	9.3608	3.9575	1.6145	9.5663	16.8698
Father education_2_	27.2565	7.6337	13.1992	27.1539	42.5586
Place of residence_1_	17.3794	6.6762	3.5911	17.4256	30.6240
Sex of household head_1_	11.2210	4.6675	1.9502	11.2886	20.5232
Child sex_1_	-8.7847	3.6059	-15.6807	-8.8233	-1.5808
Sources of drinking water_1_	-8.6198	3.9452	-16.5138	-8.5380	-0.6840
Had diarhea recently_1_	-22.4209	4.5101	-31.3079	-22.3400	-14.0301
Ever had vacination_1_	9.5261	4.4431	1.0386	9.6030	17.8134
Mother drug experience_1_	16.3906	7.9207	1.3048	16.4173	31.7264
Wealth index_1_	11.2715	4.8309	1.6908	11.3099	21.0343
Wealth index_2_	23.2884	5.0696	13.8812	23.2355	33.6861
Age of household head_1_	-7.3165	4.2327	-15.8293	-7.3215	0.8992
Birth order_1_	-12.2064	4.1476	-20.3436	-12.1372	-4.1052
Preceding birth interval_2_	10.2030	4.3467	1.6414	10.2572	18.9704
Duration of breast feeding_1_	27.4541	4.6865	17.9296	27.5496	37.0460
Child’s size_1_	23.0011	4.1485	15.2364	23.0393	31.2940
Child’s size_2_	30.2857	4.5031	21.0831	30.3849	39.4592

Since the 95% credible interval do not include zero, father’s education level, place of residence (rural), sex of the head of household (male), child’s sex (female), source of water (not improved), diarrhea (had diarrhea), drug (never took drug for intestinal parasites during pregnancy), children wealth index, birth order, preceding birth interval, duration of breast feeding and size of child at birth were found statistically significant at 5% significance level. But, age of household was found statistically insignificant.

Figure 5 displays nonlinear effects and estimated functions of mother’s age at birth in year, child’s age in month and mother’s body mass index for under five years old child data. The shaded region represents twice the point wise asymptotic standard errors of the estimated curve.

The panels in Fig. 5 show an interval marked as HDI, which stands for highest density interval. Points inside an HDI have higher probability density (credibility) than points outside the HDI, and the points inside the 95% HDI include 95% of the distribution. Thus, the 95% HDI includes the most credible values of the parameter. The 95% HDI is useful both as a summary of the distribution and as a decision tool. Specifically, the 95% HDI can be used to help decide which parameter values should be deemed not credible, that is, rejected. This decision process goes beyond probabilistic Bayesian inference, which generates the complete posterior distribution, not a discrete decision regarding which values can be accepted or rejected. One simple decision rule is that any value outside the 95% HDI is rejected. In particular, if we want to decide whether the regression coefficients are nonzero, we consider whether zero is included in the 95% HDI.

In the present study, all continuous variables shows significant effect on underweight status of children under age of five years old. Here we can see in Fig. 5, the positive and negative linear effects on malnutrition at lower level of mother’s body mass index and age of child respectively. And in addition, mother’s age at birth seems have a slight positive linear effect on the malnutrition of children.

Figure 5 showed the nonlinear effects of child’s age in month shows that the children face a risk of suffering from malnutrition during the first 30 months of their life, and then it is slight thereafter.

### Model comparison

We can use Akaike Information Criterion (AIC), Generalized Cross-Validation (GCV) and Deviance Information Criterion (DIC) as a comparative measure to choose among different models, with lower being better [14].

The core point here is to select the better model with respect to their *AIC* value. Based on Table 3, it is evident that the Bayesian linear regression model has smaller AIC value than the frequent linear model.

**Table 3:** Cumulative information for all models

Statistical Models	AIC	GCV	DIC
Frequent Gaussian linear regression model (model 1)	37599	Not available	Not available
Bayesian Gaussian linear regression model (model 2)	28741.9	Not available	3145.231
Frequent semi-parametric regression model (model 3)	Not available	9791.8	Not available
Bayesian semi-parametric regression model (model 4)	Not available	9761.2	3170.98

As illustrated the *GCV* value of semi parametric regression model in Table 3, the Bayesian approach with small value than that of the frequent approach which still is the one that can be selected.

Next, we focused on the comparison of model 1 with model 2 as well as model 2 with model 4 based on the detected results in relative to the frequent and Bayesian approach, respectively. Since model 1 and model 3 are included under the frequent approach. Since *ANOVA* function is an automatic functioning machine, we used *ANOVA* function as a comparing system of model 1 and model 3 and thus, model 3 was found to have a better fit.

As *DIC* is a criteria used as a comparing tool for Bayesian approach, model 2 and model 4 can be compared using the descripted *DIC* value in Table 3. Consequently, the models with a better fit of less *DIC* value are preferable models. Based on its performance, model 2 was chosen as a suitable model to identify the most determinants of childhood malnutrition.

## Discussions

The study aimed at examining the major influential factors behind children’s (under five year of age) malnutrition. The status of child malnutrition in the country was measured as underweight. The study showed, all Children 3115 (31.7%) were affected by malnutrition. For our study, suitably fitting (Bayesian Gaussian linear regression) model was chosen as a suitable model to identify determinants to childhood malnutrition in the Ethiopian context. The finding revealed that the covariates such as sex of child, preceding birth interval, age of child, father’s education level, source of water (the condition of an availability of water), mother’s body mass index, household head’s sex, mother’s age at birth, wealth index, birth order, diarrhea, child’s size at birth and duration of breast feeding were identified as statistically significant factors; whereas age of head of household was found to be statistically insignificant.

The results indicated that the variables such as access to health care, for children’s mothers who have not taken drug during pregnancy, had significant effects on malnutrition status of children. It was therefore implied that taking drug during pregnancy (by mothers) was more effective against underweight of children.

It is a well known fact that breast feeding had a greater influence over the growth of a child which is also confirmed by our study. Furthermore, our study revealed that diarrhea practice and duration of breast feeding also contributed significantly for children’s malnutrition which fell in line with the results recorded by Bete - Israel [20].

The living conditions along with the area of living (being in and out of an urban area) could determine the child’s malnutrition status. Problems such as poor health care access, lack of sufficiently (accessible) toilet supply, lack of modern source power like stove, cylinder and lack of awareness on the how of curing the available source of water for using it to their personal hygiene was assumed to be the risk factors of malnutrition status [21]. Our study indicated that the place of residence (rural) was associated with significant effects of malnutrition (underweight). This finding evens the finding(s) in earlier (previous) studies [22, 23]. The education attainment of fathers was also associated with significant effects to malnutrition, as of our finding. Similarly, a study [24] concluded that it (the factor in point) had an association with childhood malnutrition.

A household’s source of drinking water has been shown to be associated with malnutrition of a child in Nigeria (weight-for-age) in separate analysis [12], and that this study has also emphasized the significant of this factor of risk of malnutrition. More, it is associated with malnutrition of a child in that it impacted a risk of childhood diseases such as diarrhea, and is affective indirectly as a ‘measure of wealth’ and availability of water. This result quite consistent with some studies [11, 23, 25] but not persistent with other finding [26, 27].

Malnutrition in women is assessed using BMI. Parents with low BMI values are malnourished and are therefore likely to have undernourished and weak children. At the same time, very high BMI values indicate poor quality of the food and hence, may also imply weakness of the children [12]. The patterns of mother’s body mass index (top of Fig. 5) showed that the higher impact of BMI through the interval between 15-25, indicates that there was poor quality of food for mothers. When the BMI of non pregnant women falls below the suggested cut-off point, which is less than 18.5$\frac {kg}{m^{2}}$, malnutrition is indicated. Women who are underweight may have complications during childbirth and may deliver a child who can be underweight [6]. Our study finding indicated that there exist an association between the BMI of the mother and child’s acquiring of malnutrition. This finding is of not surprise and it correspondence with the results found by others on studies analyzing the childhood malnutrition like [23–25].

## Conclusion

Determinants that explain the cause of malnutrition in Ethiopian children community have been explored using different General additive models and Bayesian approaches. By using model comparison criteria, Gaussian linear model in Bayesian approaches was the suitable best fitted model. The findings of the present analysis indicated that sex of child, preceding birth interval, father’s education level, source of water, head of household’s sex, wealth index, birth order, diarrhea, child’s size at birth and duration of breast feeding are important determinants of childhood malnutrition. The age of child, mother’s age at birth and mother’s body mass index could also be important factors with a non linear effect for the child’s malnutrition in Ethiopia. Thus, a special emphasis need to be given on these factors to combat childhood malnutrition in developing countries.

## Additional file

Additional file 1 Histogram from Z-score value for underweight showing a normal distribution in under five years old children malnutrition, EMDHS 2014. (DOCX 35.9 kb)

## Abbreviations

AIC: Akaike information criterion
BMI: Body mass index
DIC: Deviance information criterion
EMDHS: Ethiopian mini demographic health survey
GCV: Generalized cross-validation
MCMC: Markov chain Monte Carlo
