# Supplementary material for: Bayesian Gaussian regression analysis of malnutrition for children under five years of age in Ethiopia, EMDHS 2014
Source: Arch Public Health. 2018 Mar 26;76:21. doi: 10.1186/s13690-018-0264-6 (PMC5883335; doi:10.1186/s13690-018-0264-6)
Supplement: Supplementary file 1 — Histogram from Z-score value for underweight showing a normal distribution in under five years old children malnutrition, EMDHS 2014. (DOCX 35.9 kb) [file 13690_2018_264_MOESM1_ESM.docx]

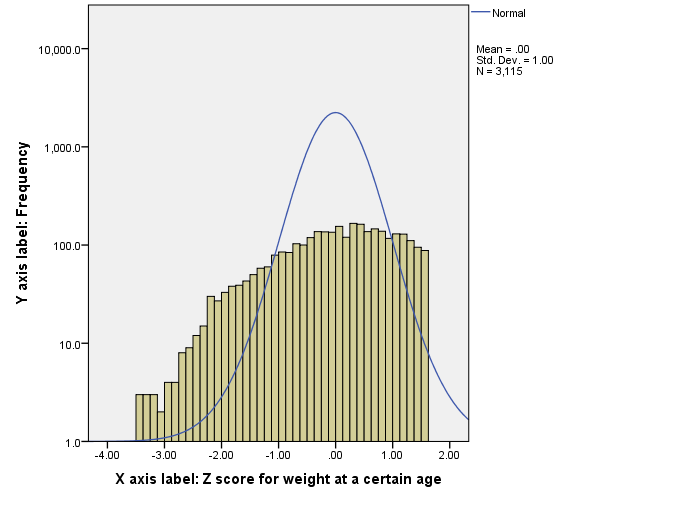


If we look closely, we will notice that the Z scores indeed have a mean zero and a standard deviation of 1. Other than that, however, Z scores follow the exact same distribution as original (observed weight at a certain age) values. That is, standardizing scores doesn’t make their distribution more “normal” in any way.

For the standard normal variable of Z score value, the region under the normal curve is (-3, 3) i.e p (-3 < Z < 3) = 0.9973, it is approximate the total area value. That means, approximately 99.73% of the measurments (essentially all) will fall within the interval equivalently (-3, 3).
